# Dr. Harold Charles Lingham

**Published:** 1912-09-14

**Authors:** 


Dr. Harold Charles Lingham has died at Herne Hill, S.E. He studied at St. Bartholomew's Hospital, and graduated in medicine, surgery, and science at the University of Edinburgh. His father was a medical practitioner at Birmingham.

